# A Genome Wide Comparison to Identify Markers to Differentiate the Sex of Larval Stages of *Schistosoma haematobium*, *Schistosoma bovis* and their Respective Hybrids

**DOI:** 10.1371/journal.pntd.0005138

**Published:** 2016-11-18

**Authors:** Julien Kincaid-Smith, Jérôme Boissier, Jean-François Allienne, Ana Oleaga, Félicité Djuikwo-Teukeng, Eve Toulza

**Affiliations:** 1 Université de Perpignan Via Domitia, IHPE UMR 5244, CNRS, IFREMER, Université de Montpellier, France; 2 Parasitology Laboratory, Instituto de Recursos Naturales y Agrobiología de Salamanca (IRNASA, CSIC), Cordel de Merinas, Spain; 3 Université des Montagnes‬ Faculty of Health Sciences, ‬Bangangté, Cameroon; University of Melbourne, AUSTRALIA

## Abstract

For scientists working on gonochoric organisms, determining sex can be crucial for many biological questions and experimental studies, such as crossbreeding, but it can also be a challenging task, particularly when no sexual dimorphism is visible or cannot be directly observed. In metazoan parasites of the genus *Schistosoma* responsible for schistosomiasis, sex is genetically determined in the zygote with a female heterogametic ZW/ZZ system. Adult flukes have a pronounced sexual dimorphism, whereas the sexes of the larval stages are morphologically indistinguishable but can be distinguished uniquely by using molecular methods. Therefore, reliable methods are needed to identify the sex of larvae individuals. Here, we present an endpoint PCR-based assay using female-specific sequences identified using a genome-wide comparative analysis between males and females. This work allowed us to identify sex-markers for *Schistosoma haematobium* and *Schistosoma bovis* but also the hybrid between both species that has recently emerged in Corsica (France). Five molecular sex-markers were identified and are female-specific in *S*. *haematobium* and the hybrid parasite, whereas three of them are also female-specific in *S*. *bovis*. These molecular markers will be useful to conduct studies, such as experimental crosses on these disease-causing blood flukes, which are still largely neglected but no longer restricted to tropical areas.

## Author Summary

Current global changes (environmental and anthropogenic) are expected to promote the spread and transmission of infectious diseases. One of the direct consequences of such changes is the modification of the geographical distribution of species, enabling natural hybridization. Such hybridization is already known to occur in schistosomes, and offspring have been shown to have superior virulence and invasive capacities. The recent outbreak of a hybrid between the human- and animal- infecting schistosomes, *S*. *haematobium* x *S*. *bovis*, in Europe (Corsica, France) clearly demonstrates this invasive capacity and raises the risk of zoonotic transmission. Therefore, it is important to study such hybrids, and experimental crosses are critical to address this issue. Here, we developed molecular sex markers for *S*. *haematobium* and *S*. *bovis* in order to distinguish gender and to be able to generate differentially introgressed hybrids, allowing us to investigate parasite fitness.

## Introduction

Flatworm parasites of the genus *Schistosoma* are well known trematodes for their serious threat to human and animal health. These blood flukes cause schistosomiasis, a neglected disease which ranks second to malaria, in terms of morbidity and mortality [1]. More than 200 million people are infected worldwide, and the parasite is endemic to 78 countries in tropical and subtropical areas [2,3]. Schistosomes are endoparasites with a complex lifecycle involving two obligatory hosts. One is a freshwater mollusc, as an intermediate host, and humans or mammals represent definitive hosts. In the definitive host, monogamous couples of worms sexually reproduce, and females release hundreds of eggs daily. The elimination of eggs through host faeces or urine in a freshwater environment allows the development of free-swimming larvae called miracidia. These larvae actively search for their intermediate mollusc hosts, which they penetrate and develop via asexual multiplication to produce thousands of vertebrate infecting larvae (cercariae). A unique feature of the *Schistosoma* genus amongst other hermaphroditic trematodes is their gonochorism. Sexes are genetically determined during egg fertilization by a ZZ/ZW chromosomal system, where the female is the heterogametic sex [4]. Sexual dimorphism strongly characterises schistosome adult worms (*i*.*e*. a muscular adult male *vs*. a thin adult female). Furthermore, females do not fully mature in the absence of males. By contrast, male and female larval stages are morphologically indistinguishable [5,6].

Recently, an outbreak of urogenital schistosomiasis occurred in Corsica [7,8]. This French Mediterranean Island is particularly attractive for tourists, and its population increases from 300,000 to 3 million people in summer season. With more than 100 people infected in Corsica, this outbreak demonstrates how easily a tropical disease, possibly under the influence of global changes (*i*.*e*. climate change and human migration), could emerge in temperate continental areas [9]. Urogenital schistosomiasis is usually caused by *S*. *haematobium*. This species is widely present in Africa and in the Arabic Peninsula, and is the only schistosome species in the vessels of the urogenital tract (mainly bladder) of humans. Surprisingly, molecular investigations (sequencing of the mitochondrial *cox*1 gene and nuclear ribosomal internal transcribed spacer DNA = ITS) have revealed that the parasite incriminated in the Corsican outbreak was not a pure *S*. *haematobium* parasite, but a hybrid between *S*. *haematobium* and *S*. *bovis* [10,11]. *S*. *bovis* is a parasite of the same monophyletic group referred to as the *S*. *haematobium* group [12]. However it is not a human, but rather a livestock parasite, infecting cows, sheep or goats, and lives in the mesentery instead of the bladder vessels. This hybrid parasite makes the epidemiological situation complex and raises the risk of zoonotic transmission. One relevant question is the importance of the hybrid vigour of a parasite in such situation. Parasite hybridization has been shown to impact parasite infectivity, virulence, transmission and/or host specificity in fungal, viral, bacterial and parasitic helminths (see [13] for recent synthesis). Such hybrid vigour has already been demonstrated in the laboratory, with *S*. *haematobium x S*. *intercalatum* (a parasite of human belonging to the same *S*. *haematobium* group) crosses. These last hybrids have a better capacity to invade and develop into their respective molluscan or experimental rodent hosts compared with both parental species [14]. Hybrid vigour of the parasite is therefore an important aspect to study, and experimental crosses are critical to explore the hybridisation process. Such experimental crosses necessitate controlling the sex of cercariae proposed to experimental rodent host infection. Because larvae sex cannot be distinguished morphologically, molecular markers based on W-specific regions are crucial to identify the larval form. Such sex markers have already been developed in *S*. *mansoni* [15–18]. It has been shown previously that massive sequencing methods allow rapid comparisons of genomes and identification of sex-specific sequences in *S*. *mansoni* [18]. Therefore, we developed a robust and reliable endpoint PCR method to distinguish the sexes of *S*. *bovis* and *S*. *haematobium* at any developmental stage. The markers can also be applied efficiently to the hybrid strain recovered from Corsica.

## Materials and Methods

### Parasitological methods

Schistosome species, hosts and origin of the parasite strains used in this study are summarized in Table 1. For each strain, molluscs were individually exposed to a single miracidium of unknown sex. Twenty-eight (for *S*. *bovis*) to 45 days (for *S*. *haematobium* or the Corsican hybrid strain) after miracidium exposure, molluscs released either male or female clonal populations of cercariae. Cercariae from single molluscs were used to individually infect hamster definitive hosts. Three months after exposing hamsters to cercariae, adult worms were recovered by portal perfusion as described previously [19]. Only at this step, are male and female morphologically distinct. Individual male and female worms were stored for subsequent molecular analyses. Male and female of the hybrid strain were used for whole genome sequencing and the subsequent identification of sex markers (see below). Thereafter, these markers were tested on male and female of the three strains (Table 1). Detailed methods employed for molluscan and rodent infections were described previously [20,21].

**Table 1:** Schistosome species, hosts and origins of the parasite strains.

Schistosome species	Intermediate host	Definitive host	Origin	Year of isolation	References
*S*. *haematobium x S*. *bovis*	*Bulinus truncatus*	*Mesocricetus auratus*	Cavu River, Corsica, (France)	2014	[10,11]
*S*. *haematobium*	*Bulinus truncatus*	*Mesocricetus auratus*	Barombi Kotto Lake, (Cameroon)	2015	This study
*S*. *bovis*	*Planorbarius metidjensis*	*Mesocricetus auratus*	Villar de la Yegua-Salamanca, (Spain)	1970	[22]

### Identification of sex markers

#### Genome sequencing

Genomic DNA was separately sequenced from males and females (one pool of 10 males and one pool of 40 females) of *S*. *haematobium x S*. *bovis* hybrid adult worms (produced in rodents after recovering eggs from urine of an infected human patient) using Illumina HiSeq 2000 PE100 technology. DNA was extracted using Qiamp DNA microkit tissue protocol from Qiagen. After the lysis step, we used 4 μl (100 mg/ml) of RNase A (Qiagen) for 2 min at room temperature to digest RNA. The final elution step was performed twice with 35 μl of Tris HCl 5 mM pH 8.5. DNA quantification was performed using a Qubit^TM^ dsDNA HS Assay Kit (Invitrogen), and concentrations were estimated at 12.7 ng/μl for males and 12.8 ng/μl for females. DNA was then sent to Genome Quebec for library construction from 700 ng of genomic DNA (for each sex). A total of 192,363,250 and 186,790,310 reads were produced from males and females, respectively.

#### *In silico* analysis

The sequencing reads were filtered for quality (PHRED score < 30 for 90% of read length; Q30/90%). Then, we removed primers and adapters with the algorithm cutadapt [23]. Finally, ~144 million (75%) reads of high quality for males and females remained for subsequent analysis.

To discriminate sex-specific sequences from unbalanced genomic introgression, high quality sequencing reads were aligned to either *S*. *haematobium*, *S*. *bovis* or to a chimeric concatenate of both genomes (allowing each read, depending on its origin, to map against the more similar location in one or the other species’ genome). We used the SchistoDB *S*. *haematobium* genome [24] and the *S*. *bovis* draft assembly genome available at the Sanger institute website. The short alignment tool Bowtie2 [25] was used; mapping was performed in single-end with pre-set parameters (—sensitive -D 15 -R 2 -L 22 -i S, 1,1.15) and by restricting to uniquely mapped reads. Because the assembly of the *S*. *bovis* genome (362 Mb) is highly fragmented (>100,000 scaffolds; N50 = 7.0 kb), most effort concentrated on mapping reads to the genome of *S*. *haematobium* (385 Mb genome; 3 833 scaffolds; N50 = 307 kb).

Two independent and complementary methods were used to detect female specific sequences, with 1) a visual inspection of reads aligned to the genomes of parental species (example in S1 Fig), and 2) a copy number variation detection using CNV-seq between male and female reads [26]. The visual inspection of genomic read alignments was performed using the Integrative Genomics Viewer (IGV) [27] to find regions showing exclusive coverage in females. Scaffolds and contigs were randomly inspected in the genomes of *S*. *bovis* and *S*. *haematobium* for potential female specific sequences. The CNV-seq method was used to compare coverage between males and females along the genome. The minimum window size was set to 500 bp, log2 ratio to 0.6 and p-value stringency to 10^−6^. *S*. *bovis* and *S*. *haematobium* reference genomes were used, alternatively, and regions showing specific coverage for females were selected.

Consensus candidate sequences were extracted using IGV, and primers were designed using the program Primer3Plus [28]. In order to confirm specificity, primers were compared (by BLAST) against the genome that they were originally identified in.

### Validation of sex markers

DNAs of single adult worms of each sex were extracted, as recommended from the QIAGEN QIAamp DNA Micro Kit protocol for isolation of genomic DNA from tissues. The final elution was in 40 μl of buffer AE (10 mM Tris·Cl, 0.5 mM EDTA, pH 9.0). Sex markers were also tested on cercariae of *S*. *haematobium*. DNA extraction from cercariae was conducted as published by our colleagues [29].

**Fig 1:**
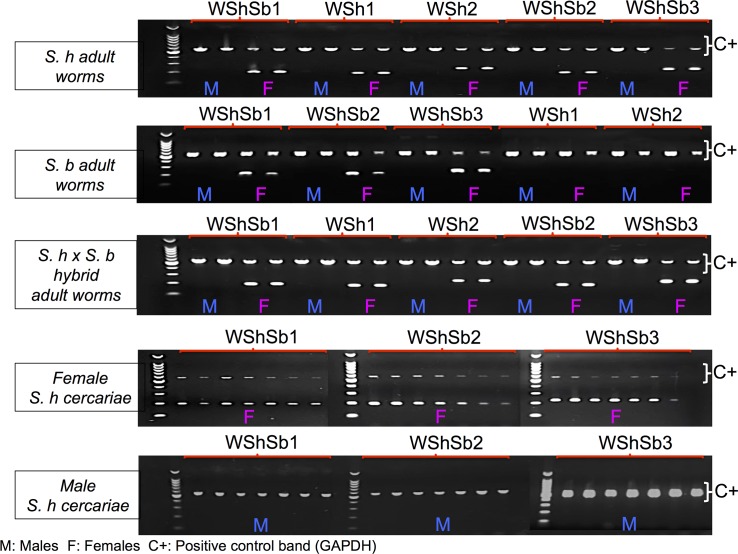
Diagnostic PCR assay of female-specific markers in males and females *Schistosoma haematobium* (*S*. *h*), *Schistosoma bovis* (*S*. *b*) and their hybrids. The first three gels correspond to PCR amplifications on adult worms (biological duplicates for males and females) of the 5 female specific markers identified with our *in silico* approach. Note that upper-bands correspond to the *GAPDH* gene control (558 bp), whereas lower bands correspond to female-specific amplification (see Table 2 for amplicon sizes). Note also that amplification is not effective for amplification from the genome of *S*. *bovis* using the two markers WSh1 and Wsh2. The last two gels show sex-specific amplification for the three markers, efficient in both species on a batch of cercariae from molluscs mono-miracidially infected with *S*. *haematobium*.

We used a multiplex approach with the *GAPDH* gene as an internal PCR control (Table 2) The reactions were carried out in a total volume of 12.5 μl containing GoTaq Flexi Reaction Buffer (Promega), 1.5 mM of MgCl_2_, 0.4 μM of each primers of both markers (at 10 μM each), 0.2 μM of dNTP solution (at 10 μM each), 0.5 U of GoTaq G2 Hot Start Polymerase (Promega, USA) and 10 ng of DNA template (except no-DNA controls). The PCR protocol was: an initial denaturation phase at 95°C for 5 min, followed by 35 cycles at 95°C for 30s, 57°C for 30s, 72°C for 40s, and a final extension at 72°C for 10 min. PCR products were examined on 1.5% agarose gels using a 100 bp-DNA Ladder (Promega) for size estimation. PCR amplifications were performed using biological duplicates of adult male and female worms of *S*. *haematobium*, *S*. *bovis* and the hybrid, and using seven replicates of a pool of 5 cercariae recovered from molluscs infected by only one miracidium (*i*.*e*. one sex). The sex of the clonal populations of cercariae was confirmed by infecting hamsters, and morphological examination of adults that developed. In total, 35 individuals were used to validate sex markers (Fig 1 and S2 Fig). Female-specific amplicons were sent for sequencing (Genoscreen, Lille, France) for each target species.

**Table 2:** Summary information of the sex-specific markers identified.

Name	Primer sequences (5’-3’)	Scaffold	Position	Amplicon size	Female specificity		
					*S*. *h*	*S*. *b*	*S*.*h x S*.*b*
GAPDH	Forward	KL251991 (*S*. *haematobium*) SBOI.contig.33617.2504 (*S*. *bovis*)	15,936–16,493	558 bp	No	No	No
	CGACCATTGATGCAGCTAAA						
	Reverse		1,000–1,557				
	TTCCAAAATCCCCTTCATTG						
WSh1	Forward	KL253191	39–225	187 bp	Yes	NA	Yes
	GCGTTCCGTTTAAAACATCG						
	Reverse						
	GTCCATGTGAGGGAATTTCG						
WSh2	Forward	KL252440	84–322	239 bp	Yes	NA	Yes
	GAATCGATGACACTGGCGTA						
	Reverse						
	CCACTGTCCTTCGGAATTGT						
WShSb1	Forward	KL252782	3,138–3,334	197 bp	Yes	Yes	Yes
	CCACTAGAGTCGTCGTCGTG						
	Reverse						
	GCTGCCGAATCCATAACAAA						
WShSb2	Forward	KL252782	8,258–8,487	230 bp	Yes	Yes	Yes
	GTTGAAATTCGCTGCTGGAT						
	Reverse						
	AATGGTTTTGGACGGAATTG						
WShSb3	Forward	KL252440	18,445–18,637	193 bp	Yes	Yes	Yes
	GGTGGTCAGGCATTGATTCT						
	Reverse						
	CATGTTTAGGCGCTTCAGGT						

NA: no amplification. *S*. *h*: *Schistosoma haematobium*. *S*. *b*: *Schistosoma bovis*

### Real-time quantitative PCR to test the efficiency of primers

Reactions were performed on pools of 3 females of *Schistosoma haematobium* or *Schistosoma bovis* using technical triplicates. The reaction mixture of a final volume of 10 μl consisted of standard reaction mix (Takyon SYBR assays; 1x), 1 μM of each primer and DNA template. The cycling protocol was: an initial denaturation step at 95°C for 5 min, followed by 40 cycles of denaturation (95°C for 10 s), annealing (56°C for 20 s) and extension (72° C for 25 s). Primer efficiencies was calculated using the slope of the standard curve on serial dilutions from 100 ng to 0.4 ng with efficiency = -1 + 10^(-1/slope)^.

### Ethics Statement

All experiments on hamsters were carried out according to national ethical standards established in the writ of February 1st, 2013 (NOR: AGRG1238753A), setting the conditions for approval, planning and operation of establishments, breeders and suppliers of animals used for scientific purposes and controls. The French Ministry of Agriculture and Fishery (Ministère de l’Agriculture et de la Pêche), and the French Ministry for Higher Education, Research and Technology (Ministère de l’Education Nationale de la Recherche et de la Technologie) approved the experiments carried out for this study and provided permit A66040 for animal experimentation. The investigator possesses the official certificate for animal experimentation delivered by both ministries (Décret n° 87–848 du 19 octobre 1987; number of the authorization 007083).

## Results

Sixty primer couples were designed and tested in this study. None of the primers designed on genome of *S*. *bovis* gave significant sex-specific results. Concerning primers designed on *S*. *haematobium* genome, we identified 5 female specific sequences for pure *S*. *haematobium* as well as for the hybrid form of the parasite, 3 of which can also discriminate female from male individuals in *S*. *bovis* (Table 2, Fig 1, and S2 Fig). Three different scaffolds of *S*. *haematobium* genome were identified as being at least in part restricted to females and thus W-chromosome specific (KL252782, KL253191, KL252440). The *GAPDH* gene (control) was retrieved from both *S*. *haematobium* and *S*. *bovis* genomes, whereas none of the sex-specific markers were detected in the *S*. *bovis* draft assembly. The results of PCR amplification of these markers are presented in Fig 1. The correspondence between the sexes of *S*. *haematobium* cercariae determined by PCR, and the observation of these parasites as adult worms following hamster perfusion confirmed the reliability of our approach. Furthermore, we tested the efficiencies of our primers using real-time quantitative PCR, and obtained efficiencies around 100% for all primer couples with a single peak in melting curves. DNA amplification was still detectable below 30 Ct for 1.5 ng of DNA template for all markers. The sequenced amplicons (S1 File) were always identical between *S*. *haematobium* and the hybrid from Corsica. The sequences were highly conserved with *S*. *bovis* (100% identical for WShSb3, 97% and 97,4% for WShSb1 and WShSb2, respectively (S2 File).

## Discussion

In the present work, we used massive sequencing data from a field *S*. *haematobium* x *S*. *bovis* hybrid schistosome strain, allowing us to successfully identify 5 female-specific markers on 3 different scaffolds from the draft genome of *S*. *haematobium*, thus corresponding to previously unplaced W-specific regions. No sex marker candidates were confirmed by PCR when they were selected based on reads aligned against the genome of *S*. *bovis*. Furthermore, we could not locate the female-specific markers originating from the *S*. *haematobium* draft genome in the *S*. *bovis* genome. This can be explained by the relatively poor quality of the present genome for *S*. *bovis*, which emphasises the need to improve the assembly. Despite this, among 5 female-specific sex markers in *S*. *haematobium*, 3 can also be used for *S*. *bovis*. The conservation of three of the markers for *S*. *bovis* females was expected due to the close relatedness of both species, which are in the same monophyletic group [12]. The two markers, Wsh1 and Wsh2, did not amplify either female or male in *S*. *bovis*. All of the markers are functional for the *S*. *haematobium x S*. *bovis* hybrid and identical to the *S*. *haematobium* sequence, which is consistent with current work in our laboratory that suggests higher levels of introgression of *S*. *haematobium* than *S*. *bovis* in the hybrid schistosome from Corsica.

We first tested existing molecular sex markers designed for *S*. *mansoni* [18] on *S*. *bovis* and *S*. *haematobium* parasites, but no amplification was achieved. The sex-specific markers identified in the present study were also tested on 2 males and 2 females *S*. *mansoni*, and could not amplify any sequence. *S*. *mansoni* does not belong to the *S*. *haematobium* group but to another monophyletic group of *Schistosoma* (referred to as *S*. *mansoni* group) [12]. The fact that sex chromosome structure, inferred by C-banding method, is known to be different among schistosome species may explain the absence of cross-group amplification [4]. Further investigations are also needed to test whether these sex markers are applicable to other species of the *S*. *haematobium* group. Indeed, the *Schistosoma* genus harbours a high diversity of species, and the *S*. *haematobium* group is the most diverse and includes 3 species that infect humans (i.e. *S*. *haematobium*, *S*. *intercalatum*, and *S*. *guineensis*) and 6 species infecting animals (*S*. *bovis*, *S*. *curassoni*, *S*. *kisumuensis*, *S*. *leiperi*, *S*. *margrebowiei* and *S*. *mattheei*), mainly ruminants and/or rodents. Interestingly, species of the *S*. *haematobium* group have frequently been incriminated in hybridization phenomena. Natural hybrids have been observed between schistosome species infecting humans (i.e. *S*. *haematobium x S*. *intercalatum* [30,31], *S*. *haematobium x S*. *mansoni* [32], *S*. *haematobium x S*. *guineensis* [33,34], between schistosome species infecting animals (*S*. *bovis x S*. *curassoni* [35,36] and between both, humans and animal infecting schistosomes (*S*. *mansoni x S*. *rodhaini* [37,38], *S*. *haematobium x S*. *bovis* [11,35,39], *S*. *haematobium x S*. *mattheei* [40], *S*. *haematobium x S*. *curassoni* [35]. These last crosses are particularly important because they raise a risk of animal reservoir hosts and zoonotic transmission. A possible way of distinguishing these hybrid forms from pure parasites would be to use both mitochondrial (*cox*1) and nuclear (e.g., ITS) markers. In addition, being able to rely on sex-specific markers for such species will enable the identification of sex of the clonal cercariae pool proposed for hamster infection, and therefore will offer the opportunity to better characterise the ecological and medical impacts of schistosome hybrids. Another interesting aspect to be developed using such sex-markers would be to create a linkage map for *S*. *haematobium* and *S*. *bovis*, as has been achieved for *S*. *mansoni* [41].

Finally, the sex markers we have identified might be used to study sex-specific population structures of schistosome larvae in the field. Such sex specific structures with males being more randomly distributed than females has been observed for *S*. *mansoni* [42]. Our sex markers would allow the testing of bias dispersal in larval populations of *S*. *haematobium* or of *S*. *bovis*.

To conclude we demonstrate throughout this work that comparing male and female genomic data is relatively straightforward and could offer a reliable way of identifying sex-specific sequences. These markers are essential for experimental crosses and to design appropriate protocols for studying schistosome species within the *S*. *haematobium* group which are currently of great concern in a context of disease emergence both in Africa and Europe.

## Supporting Information

S1 Fig Visualisation with IGV of reads aligned against *S*. *haematobium* scaffold KL252782. - Female (a) and male (b) reads aligned against *S*. *haematobium x S*. *bovis* concatenated genome using pre-set parameters.
- Female (c) and male (d) reads aligned against *S*. *haematobium x S*. *bovis* concatenated genome using unique read alignment.
Female specific sequences are easily recognisable and selected regions (red) were confirmed with our end-point PCR approach.
(TIF)

S2 Fig Diagnostic PCR assay of female-specific markers in additional males and females individuals of *Schistosoma haematobium*, and *Schistosoma bovis* adult worms. The top panel corresponds to PCR amplifications of female-specific markers from 6 females and 5 males of *Schistosoma haematobium*. All five sex markers are efficient in distinguishing male from female individuals. The bottom panel corresponds to PCR amplification of the 3 sex markers also efficient in *Schistosoma bovis*. Five males and 5 females were readily distinguished using primers WShSb1, WShSb2 and WShSb3. Note that the upper bands correspond to the *GAPDH* gene control (558 bp), whereas lower bands correspond to female-specific amplifications (see Table 2 for amplicon sizes).
(TIF)

S1 File Fasta format sequences of the female-specific amplicons in each target species (ref: *Schistosoma haematobium* reference genome, Sh: *Schistosoma haematobium*, Sb: *Schistosoma bovis*, hyb: hybrid from Corsica). (FASTA)

S2 File Multiple sequence alignment of female-specific amplicons (ref: *Schistosoma haematobium* reference genome, Sh: *Schistosoma haematobium*, Sb: *Schistosoma bovis*, hyb: hybrid from Corsica). (DOC)
