# Supplementary figures and images for: A Genome Wide Comparison to Identify Markers to Differentiate the Sex of Larval Stages of Schistosoma haematobium, Schistosoma bovis and their Respective Hybrids
Source: PLoS Negl Trop Dis. 2016 Nov 18;10(11):e0005138. doi: 10.1371/journal.pntd.0005138 (PMC5115654; doi:10.1371/journal.pntd.0005138)

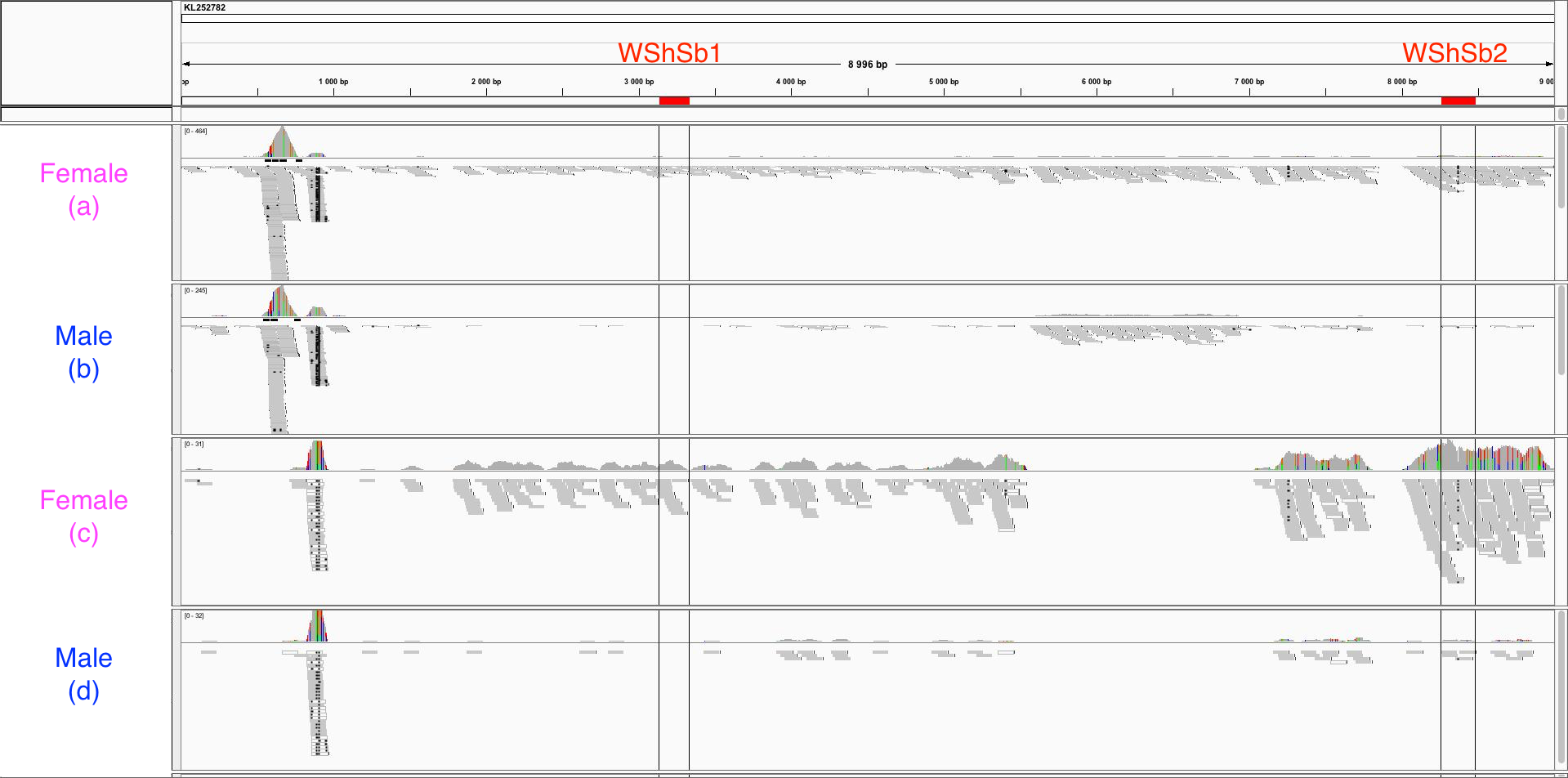

Supplement: S1 Fig — - Female (a) and male (b) reads aligned against S. haematobium x S. bovis concatenated genome using pre-set parameters. - Female (c) and male (d) reads aligned against S. haematobium x S. bovis concatenated genome using unique read alignment. Female specific sequences are easily recognisable and selected regions (red) were confirmed with our end-point PCR approach. (TIF) [file pntd.0005138.s001.tif]

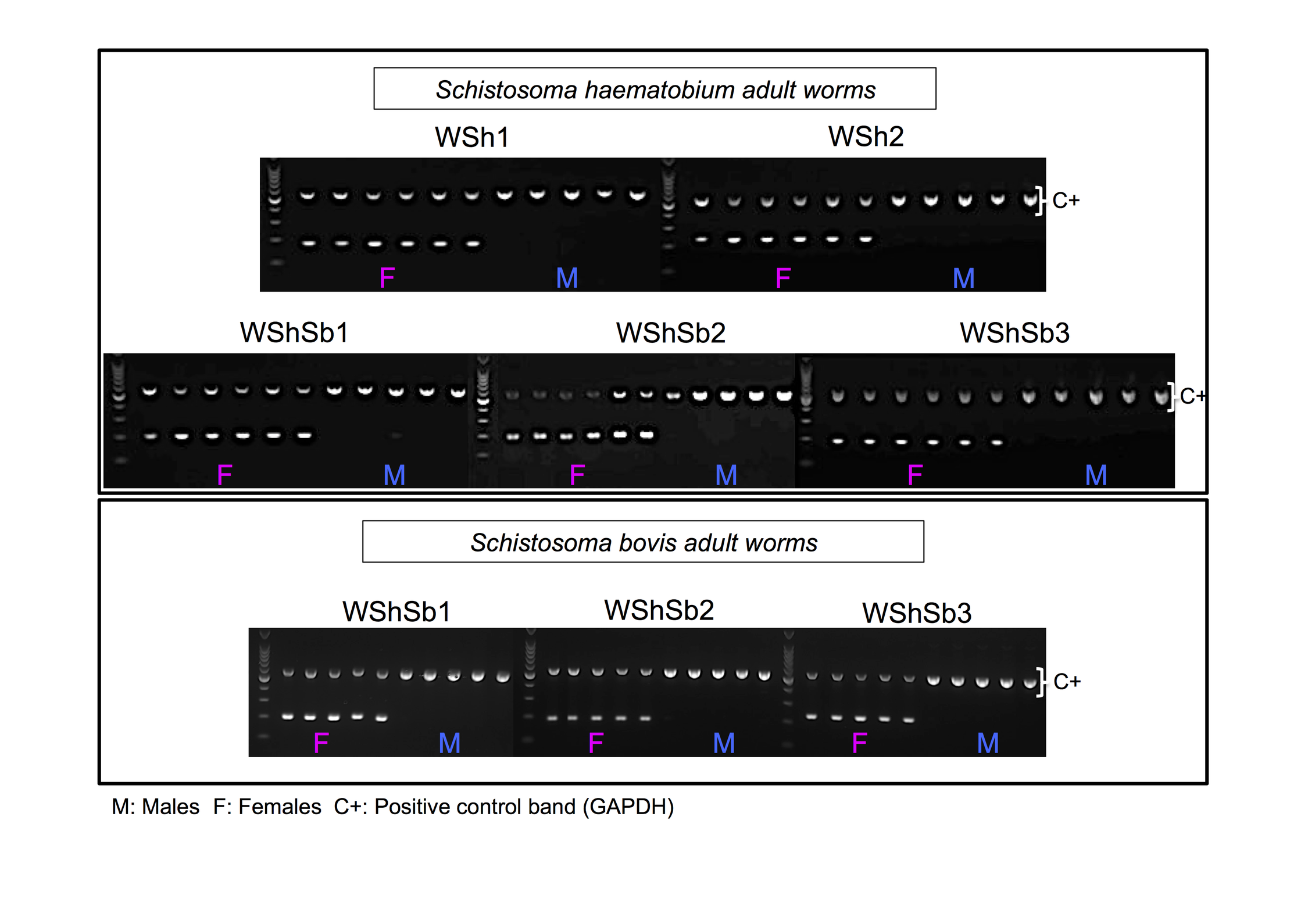

Supplement: S2 Fig — The top panel corresponds to PCR amplifications of female-specific markers from 6 females and 5 males of Schistosoma haematobium. All five sex markers are efficient in distinguishing male from female individuals. The bottom panel corresponds to PCR amplification of the 3 sex markers also efficient in Schistosoma bovis. Five males and 5 females were readily distinguished using primers WShSb1, WShSb2 and WShSb3. Note that the upper bands correspond to the GAPDH gene control (558 bp), whereas lower bands correspond to female-specific amplifications (see Table 2 for amplicon sizes). (TIF) [file pntd.0005138.s002.tif]
