# Supplementary material for: A Genome Wide Comparison to Identify Markers to Differentiate the Sex of Larval Stages of Schistosoma haematobium, Schistosoma bovis and their Respective Hybrids
Source: PLoS Negl Trop Dis. 2016 Nov 18;10(11):e0005138. doi: 10.1371/journal.pntd.0005138 (PMC5115654; doi:10.1371/journal.pntd.0005138)
Supplement: S2 File — (DOC) [file pntd.0005138.s004.doc]

1 60

WSh1_ref GCGTTCCGTT TAAAACATCG AGAATATATT TCGCTTTCTA AGTTTATTTA AAATAAATGT

WSh1_Sh GCGTTCCGTT TAAAACATCG AGAATATATT TCGCTTTCTA AGTTTATTTA AAATAAATGT

WSh1_hybri GCGTTCCGTT TAAAACATCG AGAATATATT TCGCTTTCTA AGTTTATTTA AAATAAATGT

Consensus GCGTTCCGTT TAAAACATCG AGAATATATT TCGCTTTCTA AGTTTATTTA AAATAAATGT

61 120

WSh1_ref AATTTAATTG TTTCGAAGTT TTCATAGATC ACTTCATTTG GACTAGAAAA CAGAAGGCCA

WSh1_Sh AATTTAATTG TTTCGAAGTT TTCATAGATC ACTTCATTTG GACTAGAAAA CRGAAGGCCA

WSh1_hybri AATTTAATTG TTTCGAAGTT TTCATAGATC ACTTCATTTG GACTAGAAAA CRGAAGGCCA

Consensus AATTTAATTG TTTCGAAGTT TTCATAGATC ACTTCATTTG GACTAGAAAA CrGAAGGCCA

121 180

WSh1_ref TCAAATGCCC TGGTACGGTC GAGGGTGGGG AGAGTCATTC CCCCTCTCGA AATTCCCTCA

WSh1_Sh TCAAATGCCC TGGTACGGTC GAGGGTGGGG AGAGTCATTC CCCCTCTCGA AATTCCCTCA

WSh1_hybri TCAAATGCCC TGGTACGGTC GAGGGTGGGG AGAGTCATTC CCCCTCTCGA AATTCCCTCA

Consensus TCAAATGCCC TGGTACGGTC GAGGGTGGGG AGAGTCATTC CCCCTCTCGA AATTCCCTCA

181

WSh1_ref CATGGAC

WSh1_Sh CATGGAC

WSh1_hybri CATGGAC

Consensus CATGGAC

1 60

WSh2_ref GAATCGATGA CACTGGCGTA GGTGTATACA GAATTTATCT TTCCTTCTTT TTTATATATT

WSh2_Sh GAATCGATGA CACTGGCGTA GGTGTATACA GAATTTATCT TTCCTTCTTT TTTATATATT

WSh2_hybri GAATCGATGA CACTGGCGTA GGTGTATACA GAATTTATCT TTCCTTCTTT TTTATATATT

Consensus GAATCGATGA CACTGGCGTA GGTGTATACA GAATTTATCT TTCCTTCTTT TTTATATATT

61 120

WSh2_ref ACTATCAGTG AAGTAACTAT TACTATGAAT TTAGTGTTTG TTATTTTGTG TTAATGAAGT

WSh2_Sh ACTATCAGTG AAGTAACTAT TACTATGAAT TTAGTGTTTG TTATTTTGTG TTAATGAAGT

WSh2_hybri ACTATCAGTG AAGTAACTAT TACTATGAAT TTAGTGTTTG TTATTTTGTG TTAATGAAGT

Consensus ACTATCAGTG AAGTAACTAT TACTATGAAT TTAGTGTTTG TTATTTTGTG TTAATGAAGT

121 180

WSh2_ref GTGGCAACCT GAACCTAAAA TGTAGCTGAC AGACAGACTG ACATGGGTCT CACCGGATGA

WSh2_Sh GTGGCAACCT GAACCTAAAA TGTAGCTGAC AGACAGACTG ACATGGGTCT CACCGGATGA

WSh2_hybri GTGGCAACCT GAACCTAAAA TGTAGCTGAC AGACAGACTG ACATGGGTCT CACCGGATGA

Consensus GTGGCAACCT GAACCTAAAA TGTAGCTGAC AGACAGACTG ACATGGGTCT CACCGGATGA

181 239

WSh2_ref AACCATGGAG AAACAGGTAG ATCATATTTG TATCAATAAA CAATTCCGAA GGACAGTGG

WSh2_Sh AACCATGGAG AAACAGGTAG ATCATATTTG TATCAATAAA CAATTCCGAA GGACAGTGG

WSh2_hybri AACCATGGAG AAACAGGTAG ATCATATTTG TATCAATAAA CAATTCCGAA GGACAGTGG

Consensus AACCATGGAG AAACAGGTAG ATCATATTTG TATCAATAAA CAATTCCGAA GGACAGTGG

1 60

WShSb1_ref CCACTAGAGT CGTCGTCGTG TATTGGGGTT AATAAATCAT CACCTATACC AGTCTTTGTG

WShSb1_Sh CCACTAGAGT CGTCGTCGTG TATTGGGGTT AATAAATCAT CACCTATACC AGTCTTTGTG

WShSb1_hyb CCACTAGAGT CGTCGTCGTG TATTGGGGTT AATAAATCAT CACCTATACC AGTCTTTGTG

WShSb1_Sb CCACTAGAGT CGTCGTCGTG TTTTGGGGTT AATAAATCAT CCCCTATACC AGTCTTTGTG

Consensus CCACTAGAGT CGTCGTCGTG TaTTGGGGTT AATAAATCAT CaCCTATACC AGTCTTTGTG

61 120

WShSb1_ref TTCTTGGGCA GGGGCAATGT TATGTATGCT CTTTGTAGGC AAGAACCATG CGAAGATGTC

WShSb1_Sh TTCTTGGGCA GGGGCAATGT TATGTATGCT CTTTGTAGGC AAGAACCATG CGAAGATGTC

WShSb1_hyb TTCTTGGGCA GGGGCAATGT TATGTATGCT CTTTGTAGGC AAGAACCATG CGAAGATGTC

WShSb1_Sb TTCTTGGGCA GAGGCAATGT TATGTATTCT CTTTGTCGGC AGGAACCATG CGAAGATGTC

Consensus TTCTTGGGCA GgGGCAATGT TATGTATgCT CTTTGTaGGC AaGAACCATG CGAAGATGTC

121 180

WShSb1_ref GTACGACTAG TGGAATGCTT TAAAGATATA ATGTTCGGTA CTTATGATAC ACGCACTTTT

WShSb1_Sh GTACGACTAG TGGAATGCTT TAAAGATATA ATGTTCGGTA CTTATGATAC ACGCACTTTT

WShSb1_hyb GTACGACTAG TGGAATGCTT TAAAGATATA ATGTTCGGTA CTTATGATAC ACGCACTTTT

WShSb1_Sb GTACGACTAG TGGAATGCTT TAAAGATATA ATGTTCGGTA CTTATGATAC ACGCACTTTT

Consensus GTACGACTAG TGGAATGCTT TAAAGATATA ATGTTCGGTA CTTATGATAC ACGCACTTTT

181 197

WShSb1_ref GTTATGGATT CGGCAGC

WShSb1_Sh GTTATGGATT CGGCAGC

WShSb1_hyb GTTATGGATT CGGCAGC

WShSb1_Sb GTTATGGATT CGGCAGC

Consensus GTTATGGATT CGGCAGC

1 60

WShSb2_ref GTTGAAATTC GCTGCTGGAT GAGCTAGAAC AAGCGACTCT CCAGACGTCC TTTAAGAGAA

WShSb2_Sh GTTGAAATTC GCTGCTGGAT GAGCTAGAAC AAGCGACTCT CCAGACGTCC TTTAAGAGAA

WShSb2_hyb GTTGAAATTC GCTGCTGGAT GAGCTAGAAC AAGCGACTCT CCAGACGTCC TTTAAGAGAA

WShSb2_Sb GTTGAAATTC GCTGCTGGAT GAGCTAGAAC AAGCGACTCT CCAGACGTCC TTTAAGAGAA

Consensus GTTGAAATTC GCTGCTGGAT GAGCTAGAAC AAGCGACTCT CCAGACGTCC TTTAAGAGAA

61 120

WShSb2_ref GACCTGAAAT ATTCTTAAAG ACTAAGGATT GTATTTACCA TGATATATCA ATTTCTTTTT

WShSb2_Sh GACCTGAAAT ATTCTTAAAG ACTAAGGATT GTATTTACCA TGATATATCA ATTTCTTTTT

WShSb2_hyb GACCTGAAAT ATTCTTAAAG ACTAAGGATT GTATTTACCA TGATATATCA ATTTCTTTTT

WShSb2_Sb GACCTGRAAT ATTCTTAAAG ACTAAGGATT GTATTTACCA TGATATATCA AYTTCTTTTT

Consensus GACCTGaAAT ATTCTTAAAG ACTAAGGATT GTATTTACCA TGATATATCA AtTTCTTTTT

121 180

WShSb2_ref CCCCTTTCAT CGATATCATA TCTAGGTTCC TGTCTGAAAG TATTGGTGAG TCACCCTTAC

WShSb2_Sh CCCCTTTCAT CGATATCATA TCTAGGTTCC TGTCTGAAAG TATTGGTGAG TCACCGTTAC

WShSb2_hyb CCCCTTTCAT CGATATCATA TCTAGGTTCC TGTCTGAAAG TATTGGTGAG TCACCGTTAC

WShSb2_Sb CCMCTTTCAT CGATATCATA TCTAGGTTCC TGTCTGAAAG TATTGGTGAG TCACCSTTAC

Consensus CCcCTTTCAT CGATATCATA TCTAGGTTCC TGTCTGAAAG TATTGGTGAG TCACC.TTAC

181 230

WShSb2_ref TAGACACAGA AGCTTGTTTT GTAAGAGCTT CAATTCCGTC CAAAACCATT

WShSb2_Sh TAGACACAGA AGCTTGTTTT GTAAAAGCTT CAATTCCGTC CAAAACCATT

WShSb2_hyb TAGACACAGA AGCTTGTTTT GTAAAAGCTT CAATTCCGTC CAAAACCATT

WShSb2_Sb TAGAMACAGA AKCTTGTTTT GTAAAAGCTT CAATTCCGTC CAAAACCATT

Consensus TAGAcACAGA AgCTTGTTTT GTAAaAGCTT CAATTCCGTC CAAAACCATT

1 60

WShSb3_ref GGTGGTCAGG CATTGATTCT TTCATGGTTT CGATAAAATT CAATGATGTA CACAGCCCTA

WShSb3_Sh GGTGGTCAGG CATTGATTCT TTCATGGTTT CGATAAAATT CAATGATGTA CACAGCCCTA

WShSb3_Sb GGTGGTCAGG CATTGATTCT TTCATGGTTT CGATAAAATT CAATGATGTA CACAGCCCTA

WShSb3_hyb GGTGGTCAGG CATTGATTCT TTCATGGTTT CGATAAAATT CAATGATGTA CACAGCCCTA

Consensus GGTGGTCAGG CATTGATTCT TTCATGGTTT CGATAAAATT CAATGATGTA CACAGCCCTA

61 120

WShSb3_ref AACTGAGAAC CATATACTTA CTTTGGTGCC TTCAAGCTCA ACTCTTCTCA AACTGCTACA

WShSb3_Sh AACTGAGAAC CATATACTTA CTTTGGTGCC TTCAAGCTCA ACTCTTCTCA AACTGCTACA

WShSb3_Sb AACTGAGAAC CATATACTTA CTTTGGTGCC TTCAAGCTCA ACTCTTCTCA AACTGCTACA

WShSb3_hyb AACTGAGAAC CATATACTTA CTTTGGTGCC TTCAAGCTCA ACTCTTCTCA AACTGCTACA

Consensus AACTGAGAAC CATATACTTA CTTTGGTGCC TTCAAGCTCA ACTCTTCTCA AACTGCTACA

121 180

WShSb3_ref AACAGCTAGA GAATCTATAT AATAGGCAGA TATTTAATCT CAATTTTGTA ATAACCTGAA

WShSb3_Sh AACAGCTAGA GAATCTATAT AATAGGCAGA TATTTAATCT CAATTTTGTA ATAACCTGAA

WShSb3_Sb AACAGCTAGA GAATCTATAT AATAGGCAGA TATTTAATCT CAATTTTGTA ATAACCTGAA

WShSb3_hyb AACAGCTAGA GAATCTATAT AATAGGCAGA TATTTAATCT CAATTTTGTA ATAACCTGAA

Consensus AACAGCTAGA GAATCTATAT AATAGGCAGA TATTTAATCT CAATTTTGTA ATAACCTGAA

181 193

WShSb3_ref GCGCCTAAAC ATG

WShSb3_Sh GCGCCTAAAC ATG

WShSb3_Sb GCGCCTAAAC ATG

WShSb3_hyb GCGCCTAAAC ATG

Consensus GCGCCTAAAC ATG
